# The European Reference Network on Genetic Tumour Risk Syndromes (ERN GENTURIS): benefits for patients, families, and health care providers

**DOI:** 10.1007/s10689-025-00457-9

**Published:** 2025-03-30

**Authors:** Manon Engels, Katarzyna Urbanczyk, Jurriaan Hölzenspies, Claas Röhl, Nicoline Geverink, Nicoline Hoogerbrugge

**Affiliations:** 1 Department of Human Genetics, Radboud university medical center, Nijmegen, The Netherlands; 2 NF Kinder, Vienna, Austria; 3 Department of Experimental Urology, Radboud university medical center, Nijmegen, The Netherlands

**Keywords:** European Reference Network, Genetic tumour risk syndrome, Hereditary cancer, Rare diseases, Cross border health care, Care, Guidelines, Patient, Education

## Abstract

The European Reference Network on Genetic Tumour Risk Syndromes (ERN GENTURIS) established in 2017 and connecting more than 50 European expert centres improves access to diagnosis, treatment, and the provision of high-quality healthcare for patients with rare genetic tumour risk syndromes (hereditary cancer), no matter where they live in Europe.

## European Reference Networks

A rare disease is defined as a condition that affects fewer than 1 in 2000 people. Around 30 million people in the European Union (EU) are affected by one of the 6000 to 8000 known rare diseases [1].

The European Reference Networks (ERNs) are cross-border networks connecting health care providers and centres of expertise in highly specialised healthcare in the EU and Norway, for the purpose of improving access to diagnosis, treatment, and the provision of high-quality and cost-effective healthcare for patients with rare, low-prevalence and complex diseases. ERNs pool expertise, knowledge and resources at the European level and enhance knowledge generation, training, and research in rare diseases. Launched in 2017 under Directive 2011/24/EU on patients’ rights in cross-border healthcare the 24 ERNs currently include 1619 specialised centres in 382 hospitals and over 300 patient representatives across 27 EU Member States and Norway [1].

## The ERN for genetic tumour risk syndromes (ERN GENTURIS)

Genetic tumour risk syndromes (genturis, hereditary cancer) are rare, low-prevalence and/or complex disorders in which inherited genetic variants strongly predispose individuals to the development of tumours, which are often diagnosed at a young age and located in multiple organ systems. However, not all genetic tumour predisposing syndromes are classified as a genturis, because some of these syndromes have characteristics that better match the inclusion criteria for one of the other ERNs. The list of genturis syndromes thus only includes syndromes that have been classified as a genturis by our expert members. While genturis are characterised by tumourigenesis, development of additional non-tumour related health issues is also frequently observed in genturis patients. Approximately 2–4% of cancers have a hereditary origin. At present, only a small minority of people with a genturis has been diagnosed as such, which hampers optimal treatment and prognosis. Many genturis patients remain unrecognized, due to the complex diagnostic criteria, rarity of the syndrome, phenotypic variability, and varying age of onset [2–4]. However, if the hereditary origin of cancer is discovered in time, risk management options can be provided to prevent a first or second cancer from occurring or to detect malignant tumours at an early stage. This is often related to a better prognosis in both patients and their family as patients with hereditary cancer can benefit from a treatment adapted to the genetic predisposition and/or phenotype [5–11] and other family members could also have inherited the genetic predisposition causing the syndrome.

ERN GENTURIS takes a family-based approach, focussing not just on the individual who first presents with the genturis, but also on their relatives who may be at high hereditary risk of cancer. Within ERN GENTURIS, the rare or low-prevalence complex syndromes are divided into four thematic disease groups (Table 1).

**Table 1:** ERN GENTURIS Thematic Disease Groups (https://www.genturis.eu/l=eng/thematic-disease-groups.html) including constitutent conditions (with hyperlinks to the information pages on the ERN GENTURIS website (www.genturis.eu)

**Thematic Group 1: Schwannomatosis and neurofibromatosis**(https://www.genturis.eu/l=eng/thematic-disease-groups/schwannomatosis-and-neurofibromatosis.html) • *NF2*-related schwannomatosis (https://www.genturis.eu/l=eng/thematic-disease-groups/schwannomatosis-and-neurofibromatosis/nf2-related-schwannomatosis.html) and mosaic *NF2*-related schwannomatosis • Non-*NF2*-related schwannomatosis (https://www.genturis.eu/l=eng/thematic-disease-groups/schwannomatosis-and-neurofibromatosis/non-nf2-related-schwannomatosis.html; *LZTR1*-related schwannomatosis, *SMARCB1*-related schwannomatosis, 22q-related schwannomatosis, mosaic schwannomatosis, schwannomatosis-not elsewhere classified, schwannomatosis-not otherwise specified) • Neurofibromatosis type 1(https://www.genturis.eu/l=eng/thematic-disease-groups/schwannomatosis-and-neurofibromatosis/neurofibromatosis-type-1.html), mosaic neurofibromatosis type 1, Neurofibromatosis-Noonan Syndrome	**Thematic Group 3: Hereditary breast and ovarian cancer syndrome**https://www.genturis.eu/l=eng/thematic-disease-groups/hereditary-breast-and-ovarian-cancer-syndrome.html • Hereditary breast and ovarian cancer syndrome (https://www.genturis.eu/l=eng/thematic-disease-groups/hereditary-breast-and-ovarian-cancer-syndrome/hereditary-breast-and-ovarian-cancer.html)
**Thematic Group 2: Lynch syndrome and polyposis **(https://www.genturis.eu/l=eng/thematic-disease-groups/lynch-syndrome-and-polyposis.html) • Hereditary nonpolyposis colorectal cancer o Lynch syndrome (https://www.genturis.eu/l=eng/thematic-disease-groups/lynch-syndrome-and-polyposis/lynch-syndrome.html) o Familial colorectal cancer Type X • Polyposis syndromes o Adenomatous polyposis syndromes (https://www.genturis.eu/l=eng/thematic-disease-groups/lynch-syndrome-and-polyposis/polyposis-syndromes.html; Familial adenomatous polyposis, Attenuated familial adenomatous polyposis including *APC*-related attenuated familial adenomatous polyposis, *AXIN2*-associated polyposis, *MUTYH*-associated polyposis, Polymerase proofreading associated polyposis, *NTHL1*-associated polyposis, *MSH3*-associated polyposis) o Hamartomatous and other polyposis syndromes (https://www.genturis.eu/l=eng/thematic-disease-groups/lynch-syndrome-and-polyposis/polyposis-syndromes.html; Peutz-Jeghers syndrome, Juvenile polyposis syndrome, Serrated polyposis syndrome, Hereditary mixed polyposis syndrome) o Gastric adenocarcinoma and proximal polyposis of the stomach	**Thematic Group 4: Other rare, predominantly malignant, genturis **(https://www.genturis.eu/l=eng/thematic-disease-groups/other-rare-genturis.html) • *PTEN* Hamartoma tumour Syndrome (https://www.genturis.eu/l=eng/thematic-disease-groups/other-rare-genturis/pten-hamartoma-tumour-syndrome.html) • heritable *TP53*-related cancer syndrome / Li-Fraumeni syndrome (https://www.genturis.eu/l=eng/thematic-disease-groups/other-rare-genturis/heritable-tp53-related-cancer-syndrome-li-fraumeni-syndrome.html) and Li-Fraumeni-like syndrome • Birt-Hogg-Dubé Syndrome (https://www.genturis.eu/l=eng/thematic-disease-groups/other-rare-genturis/birt-hogg-dube-syndrome.html) • Familial Melanoma (https://www.genturis.eu/l=eng/thematic-disease-groups/other-rare-genturis/familial-melanoma.html) including familial atypical multiple mole melanoma syndrome, Melanoma and neural system tumour syndrome • Constitutional Mismatch Repair Deficiency (https://www.genturis.eu/l=eng/thematic-disease-groups/other-rare-genturis/constitutional-mismatch-repair-deficiency.html) • Rhabdoid Tumour Predisposition Syndromes (https://www.genturis.eu/l=eng/thematic-disease-groups/other-rare-genturis/rhabdoid-tumor-predisposition-syndromes-rtps1-rtps2.html) • Diffuse Gastric and Lobular Breast Cancer Syndrome (https://www.genturis.eu/l=eng/thematic-disease-groups/other-rare-genturis/diffuse-gastric-and-lobular-breast-cancer-syndrome.html) • Carney Complex (https://www.genturis.eu/l=eng/thematic-disease-groups/other-rare-genturis/carney-complex.html) • Hereditary Papillary Renal Cell Carcinoma (https://www.genturis.eu/l=eng/thematic-disease-groups/other-rare-genturis/hereditary-papillary-renal-cell-carcinoma.html) • Ataxia-Telangiectasia (https://www.genturis.eu/l=eng/thematic-disease-groups/other-rare-genturis/ataxia-telangiectasia.html) • Bloom syndrome (https://www.genturis.eu/l=eng/thematic-disease-groups/other-rare-genturis/bloom-syndrome.html) • Naevoid basal cell carcinoma syndrome / Gorlin syndrome (https://www.genturis.eu/l=eng/thematic-disease-groups/other-rare-genturis/naevoid-basal-cell-carcinoma-syndrome-gorlin-syndrome.html) • Werner Syndrome (https://www.genturis.eu/l=eng/thematic-disease-groups/other-rare-genturis/werner-syndrome.html) • Hereditary Leiomyomatosis and Renal Cell Cancer (https://www.genturis.eu/l=eng/thematic-disease-groups/other-rare-genturis/hereditary-leiomyomatosis-and-renal-cell-cancer.html) • von Hippel-Lindau disease (https://www.genturis.eu/l=eng/thematic-disease-groups/other-rare-genturis/von-hippel-lindau-disease.html) • Fanconi anaemia (https://www.genturis.eu/l=eng/thematic-disease-groups/other-rare-genturis/fanconi-anaemia.html) • Hereditary pheochromocytoma-paraganglioma (https://www.genturis.eu/l=eng/thematic-disease-groups/other-rare-genturis/hereditary-pheochromocytoma-paraganglioma.html) • *BAP1*- related tumour predisposition syndrome • *DICER1* tumour-predisposition syndrome • *MITF*-related melanoma and renal cell carcinoma predisposition syndrome • Inherited renal cancer-predisposing syndrome, including Familial papillary thyroid carcinoma with renal papillary neoplasia, Hereditary clear cell renal cell carcinoma, Inherited cancer-predisposing syndrome due to biallelic *BRCA2* mutations • Familial papillary or follicular thyroid carcinoma (Familial nonmedullary thyroid carcinoma) • *MBD4*-related tumour predisposition syndrome • hereditary retinoblastoma • Familial atrial myxoma • Carney-Stratakis syndrome • Carney triad

This is a dynamic list of the syndromes (genturis) that fall within the remit of ERN GENTURIS based on selection by ERN GENTURIS experts. The most up-to-date version can be found on the ERN GENTURIS website (https://www.genturis.eu)

**Fig. 1:**
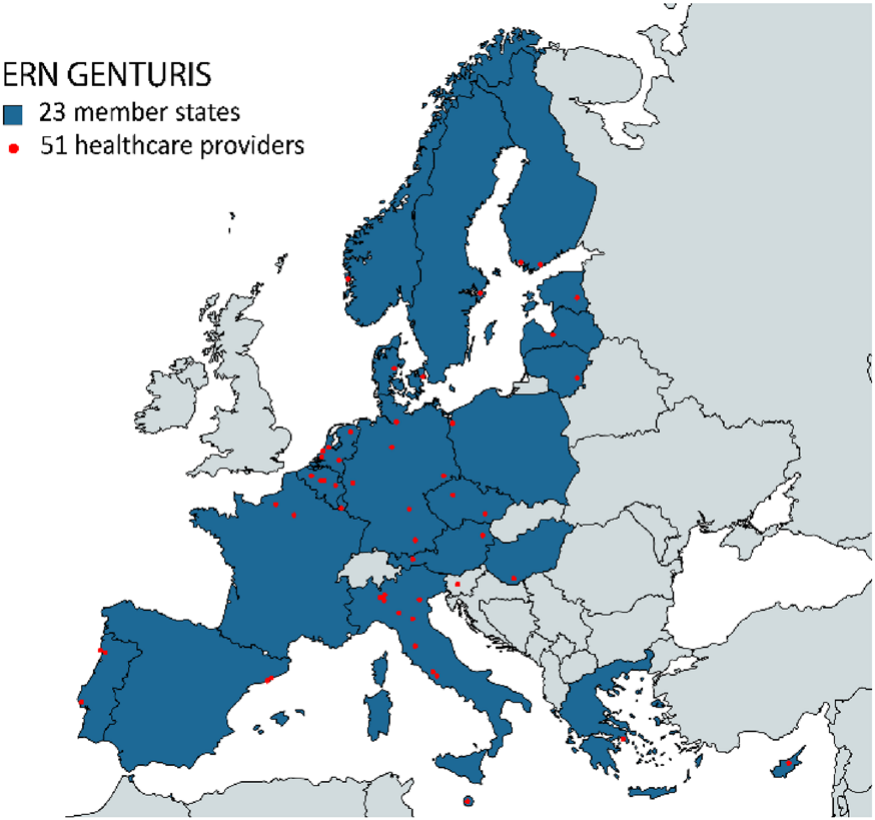
Map of Europe, indicating the countries (dark blue) and healthcare providers (red dots) that are represented in ERN GENTURIS

ERN GENTURIS comprises 51 centres in 23 European countries (Fig. 1) and 8 patient representatives and is coordinated by Radboud university medical center in Nijmegen, the Netherlands.

## Organisation of care

Since the start of ERN GENTURIS in March 2017 an average of ~ 10,000 new genturis patients per year were referred to the health care providers participating in this ERN.^1^ All ERN members have access to the Clinical Patient Management System (CPMS). The CPMS is a dedicated IT platform through which a ERN can convene ‘virtual’ advisory boards of medical specialists to discuss complex patient cases with other ERN members within and across ERNs. ERN GENTURIS CPMS meetings, organised twice per month, are the equivalent of multidisciplinary case discussions in a hospital. The type of questions that are discussed are on the level of: 1. (pathogenicity of) variants, 2. phenotype and cancer risk, and 3. prevention and treatment. The support and advice obtained during these meetings is focussed on improving care for the patient.

In case the treating physician (however not a ERN GENTURIS member) of a genturis patient based at a hospital located in the EU or an EEA Member State would like to refer a patient to be anonymously discussed by a multidisciplinary team of experts via the CPMS to obtain advice, informed consent from the patient is mandatory (Table 2).

**Table 2:** Specific links for patients, families, and health care providers to benefit from ERN GENTURIS

Topic	Link to more information
Keep informed about ERN GENTURIS activities	https://www.genturis.eu/ LinkedIn: https://www.linkedin.com/company/ern-genturis/ Subscribe to the newsletter: https://www.genturis.eu/l=eng/Home.html#GenturisNews
Becoming a member of the network	An expert centre can become a member of ERN GENTURIS by submitting an application in response to a call by the European Commission to join existing ERNs. The hospital or healthcare institute needs national endorsement and must meet several general and specific criteria https://www.genturis.eu/l=eng/our-experts/joining-ern-genturis.html
ERN GENTURIS healthcare providers	https://www.genturis.eu/l=eng/our-experts/our-healthcare-providers.html
**Organisation of care**	https://www.genturis.eu/l=eng/our-experts/members-area.html
Information for healthcare professionals and patients considering a genetic test to look for an inherited cause of cancer	https://www.cancergenetics.eu/
Discussion of complex patient cases within and across ERNs	Clinical Patient Management System: https://cpms2.ern-net.eu/screen/public If you are not a member of ERN GENTURIS you can find information on how to refer a patient on our website: https://www.genturis.eu/l=eng/our-experts/referring-a-patient.html
**Clinical guidelines and pathways**	https://www.genturis.eu/l=eng/guidelines-and-pathways.html
ERN GENTURIS guidelines	https://www.genturis.eu/l=eng/guidelines-and-pathways/clinical-practice-guidelines.html
	**Written by ERN GENTURIS** (https://www.genturis.eu/l=eng/guidelines-and-pathways/clinical-practice-guidelines/written-by-ern-genturis.html) (full guideline document, journal publication, pocket guide and plain language summary available)**:** PTEN Hamartoma Tumour Syndrome (PHTS)(https://www.genturis.eu/l=eng/guidelines-and-pathways/clinical-practice-guidelines/written-by-ern-genturis/phts-guideline.html), Li-Fraumeni and Heritable *TP53*-related cancer syndromes (https://www.genturis.eu/l=eng/guidelines-and-pathways/clinical-practice-guidelines/written-by-ern-genturis/htp53rc-guideline.html), neurofibromatosis type 1 (https://www.genturis.eu/l=eng/guidelines-and-pathways/clinical-practice-guidelines/written-by-ern-genturis/neurofibromatosis-type-1-guideline.html), schwannomatosis (https://www.genturis.eu/l=eng/guidelines-and-pathways/clinical-practice-guidelines/written-by-ern-genturis/schwannomatosis-guideline.html), Birt-Hogg-Dubé syndrome (https://www.genturis.eu/l=eng/guidelines-and-pathways/clinical-practice-guidelines/written-by-ern-genturis/bhd-syndrome-guideline.html) and constitutional mismatch repair deficiency (https://www.genturis.eu/l=eng/guidelines-and-pathways/clinical-practice-guidelines/written-by-ern-genturis/cmmrd-guideline.html)
	**Co-authored guidelines** (https://www.genturis.eu/l=eng/guidelines-and-pathways/clinical-practice-guidelines/co-authored-by-ern-genturis.html) on gastro-intestinal tumours (https://www.genturis.eu/l=eng/guidelines-and-pathways/clinical-practice-guidelines/co-authored-by-ern-genturis/gist.html), bone sarcomas (https://www.genturis.eu/l=eng/guidelines-and-pathways/clinical-practice-guidelines/co-authored-by-ern-genturis/bone-sarcomas.html), and soft tissue and visceral sarcomas (https://www.genturis.eu/l=eng/guidelines-and-pathways/clinical-practice-guidelines/co-authored-by-ern-genturis/soft-tissue-and-visceral-sarcomas.html)
	**Endorsed guidelines:** https://www.genturis.eu/l=eng/guidelines-and-pathways/clinical-practice-guidelines/endorsed-by-ern-genturis.html
ERN GENTURIS care pathways	https://www.genturis.eu/l=eng/guidelines-and-pathways/care-pathways.html
**Patient participation and patient journeys**	Patient organisations based in Europe whose disease and/or group of diseases are included in ERN GENTURIS and who would like to become a member organisation should fulfil the criteria specified in the GENTURIS ePAGs Council Terms of References, and reach out to EURORDIS which coordinates the establishment of ePAGs https://www.genturis.eu/l=eng/patient-area.html
Patient organisations	https://www.genturis.eu/l=eng/patient-area/patient-associations.html
ePAGs	https://www.genturis.eu/l=eng/patient-area/patient-advocacy-group.html
Patient journeys	https://www.genturis.eu/l=eng/guidelines-and-pathways/patient-journeys.html
**Education**	https://www.genturis.eu/l=eng/education-and-training.html
General information on the diseases included in ERN GENTURIS – website summaries, with references to documents for clinicians	Within ERN GENTURIS, the rare or complex syndromes are divided into four thematic groups (https://www.genturis.eu/l=eng/thematic-disease-groups.html): (1) Schwannomatosis and neurofibromatosis (https://www.genturis.eu/l=eng/thematic-disease-groups/schwannomatosis-and-neurofibromatosis.html)including: NF2-related schwannomatosis (https://www.genturis.eu/l=eng/thematic-disease-groups/schwannomatosis-and-neurofibromatosis/nf2-related-schwannomatosis.html) and mosaic NF2-related schwannomatosis, Non-NF2-related schwannomatosis (https://www.genturis.eu/l=eng/thematic-disease-groups/schwannomatosis-and-neurofibromatosis/non-nf2-related-schwannomatosis.html LZTR1-related schwannomatosis, SMARCB1-related schwannomatosis, 22q-related schwannomatosis, mosaic schwannomatosis, schwannomatosis-not elsewhere classified, schwannomatosis-not otherwise Specified), Neurofibromatosis type 1 (https://www.genturis.eu/l=eng/thematic-disease-groups/schwannomatosis-and-neurofibromatosis/neurofibromatosis-type-1.html), mosaic neurofibromatosis type 1 and neurofibromatosis-Noonan Syndrome,(2) Lynch syndrome and polyposis(https://www.genturis.eu/l=eng/thematic-disease-groups/lynch-syndrome-and-polyposis.html)including hereditary nonpolyposis colon cancer, Lynch syndrome (https://www.genturis.eu/l=eng/thematic-disease-groups/lynch-syndrome-and-polyposis/lynch-syndrome.html), Familial colorectal cancer Type X, adenomatous polyposis syndromes (https://www.genturis.eu/l=eng/thematic-disease-groups/lynch-syndrome-and-polyposis/polyposis-syndromes.html Familial adenomatous polyposis, Attenuated familial adenomatous polyposis including APC-related attenuated familial adenomatous polyposis, AXIN2-associated polyposis, MUTYH-associated polyposis, polymerase proofreading associated polyposis, NTHL1-associated polyposis, MSH3-associated polyposis), hamartomatous and other polyposis syndromes (https://www.genturis.eu/l=eng/thematic-disease-groups/lynch-syndrome-and-polyposis/polyposis-syndromes.html Peutz-Jeghers syndrome, Juvenile polyposis syndrome, Serrated polyposis syndrome, Hereditary mixed polyposis syndrome), gastric adenocarcinoma and proximal polyposis of the stomach (3) Hereditary breast and ovarian cancer syndrome (https://www.genturis.eu/l=eng/thematic-disease-groups/hereditary-breast-and-ovarian-cancer-syndrome.html, https://www.genturis.eu/l=eng/thematic-disease-groups/hereditary-breast-and-ovarian-cancer-syndrome/hereditary-breast-and-ovarian-cancer.html) (4) Other rare, predominantly malignant, genturis (https://www.genturis.eu/l=eng/thematic-disease-groups/other-rare-genturis.html) including >PTEN Hamartoma tumour Syndrome (https://www.genturis.eu/l=eng/thematic-disease-groups/other-rare-genturis/pten-hamartoma-tumour-syndrome.html), heritable TP53-related cancer syndrome: Li Fraumeni syndrome (https://www.genturis.eu/l=eng/thematic-disease-groups/other-rare-genturis/heritable-tp53-related-cancer-syndrome-li-fraumeni-syndrome.html) and Li-Fraumeni-like syndrome, Birt-Hogg-Dubé Syndrome (https://www.genturis.eu/l=eng/thematic-disease-groups/other-rare-genturis/birt-hogg-dube-syndrome.html), Familial Melanoma (https://www.genturis.eu/l=eng/thematic-disease-groups/other-rare-genturis/familial-melanoma.html) including familial atypical multiple mole melanoma syndrome, Melanoma and neural system tumour syndrome, Constitutional Mismatch Repair Deficiency (https://www.genturis.eu/l=eng/thematic-disease-groups/other-rare-genturis/constitutional-mismatch-repair-deficiency.html), Rhabdoid Tumour Predisposition Syndromes (https://www.genturis.eu/l=eng/thematic-disease-groups/other-rare-genturis/rhabdoid-tumor-predisposition-syndromes-rtps1-rtps2.html), Diffuse Gastric and Lobular Breast Cancer Syndrome (https://www.genturis.eu/l=eng/thematic-disease-groups/other-rare-genturis/diffuse-gastric-and-lobular-breast-cancer-syndrome.html), Carney Complex (https://www.genturis.eu/l=eng/thematic-disease-groups/other-rare-genturis/carney-complex.html), Hereditary Papillary Renal Cell Carcinoma (https://www.genturis.eu/l=eng/thematic-disease-groups/other-rare-genturis/hereditary-papillary-renal-cell-carcinoma.html), Ataxia-Telangiectasia (https://www.genturis.eu/l=eng/thematic-disease-groups/other-rare-genturis/ataxia-telangiectasia.html), Bloom syndrome (https://www.genturis.eu/l=eng/thematic-disease-groups/other-rare-genturis/bloom-syndrome.html), Naevoid basal cell carcinoma syndrome / Gorlin syndrome (https://www.genturis.eu/l=eng/thematic-disease-groups/other-rare-genturis/naevoid-basal-cell-carcinoma-syndrome-gorlin-syndrome.html), Werner Syndrome (https://www.genturis.eu/l=eng/thematic-disease-groups/other-rare-genturis/werner-syndrome.html), Hereditary Leiomyomatosis and Renal Cell Cancer (https://www.genturis.eu/l=eng/thematic-disease-groups/other-rare-genturis/hereditary-leiomyomatosis-and-renal-cell-cancer.html), von Hippel-Lindau disease (https://www.genturis.eu/l=eng/thematic-disease-groups/other-rare-genturis/von-hippel-lindau-disease.html), Fanconi anaemia (https://www.genturis.eu/l=eng/thematic-disease-groups/other-rare-genturis/fanconi-anaemia.html), Hereditary pheochromocytoma-paraganglioma (https://www.genturis.eu/l=eng/thematic-disease-groups/other-rare-genturis/hereditary-pheochromocytoma-paraganglioma.html), BAP1- related tumour predisposition syndrome, DICER1 tumour-predisposition syndrome, MITF-related melanoma and renal cell carcinoma predisposition syndrome, Inherited renal cancer-predisposing syndrome, including Familial papillary thyroid carcinoma with renal papillary neoplasia, Hereditary clear cell renal cell carcinoma, Inherited cancer-predisposing syndrome due to biallelic BRCA2 mutations, Familial papillary or follicular thyroid carcinoma (Familial nonmedullary thyroid carcinoma), related tumour predisposition syndrome, hereditary retinoblastoma, Familial atrial myxoma, Carney-Stratakis syndrome, Carney triad
Webinars	https://www.genturis.eu/l=eng/education-and-training/webinars.html
Courses	https://www.genturis.eu/l=eng/education-and-training/upcoming-trainings-and-courses.html https://www.genturis.eu/l=eng/education-and-training/past-trainings-and-courses.html
Journal round up	https://www.genturis.eu/l=eng/research/monthly-journal-round-up.html
**Research and registries**	https://www.genturis.eu/l=eng/research.html
ERN publications	https://www.genturis.eu/l=eng/research/publications.html
ERN GENTURIS research projects	https://www.genturis.eu/l=eng/research/research-projects.html
ERN GENTURIS registries	https://www.genturis.eu/l=eng/research/genturis-registry.html https://genturis-registry.eu/

### Guidelines and pathways

National healthcare systems in the EU have social objectives focussed on delivering equal, efficient, and high-quality health services at affordable costs. However, there are considerable differences in the standards of care between EU countries and even between healthcare centres in the same country. To unify care throughout Europe it is important to write European clinical practice guidelines. Clinical practice guidelines are recommendations for clinicians on diagnosis, treatment, and surveillance of patients with a specific disease, e.g. a genturis. 

For many rare diseases, assessment of clinical recommendations using a purely evidence-based approach, such as GRADE, would result in almost exclusively weak recommendations as the volume of available peer-reviewed evidence is often small and restricted to a limited number of scientific publications*.* To balance the weight of published evidence and the wealth of expert experience and knowledge, guidelines written by ERN GENTURIS include a systematic and comprehensive literature search and a modified Delphi survey. Recommendations are graded using the following scale: strong—expert consensus and consistent evidence; moderate—expert consensus with inconsistent evidence and/or new evidence likely to support the recommendation; weak—expert majority decision without consistent evidence. ERN GENTURIS guideline groups consist of experts from the multidisciplinary team and patient representatives and include representation from multiple European countries.

Presently, ERN GENTURIS has already developed 6 guidelines: *PTEN* Hamartoma Tumour Syndrome, Li-Fraumeni and Heritable *TP53*-related cancer syndromes, neurofibromatosis type 1, schwannomatosis, Birt-Hogg-Dubé syndrome and constitutional mismatch repair deficiency. In addition to a peer-reviewed journal publication, ERN GENTURIS develops a more detailed full guideline document without word or reference count restriction, a pocket guide (summary of the guideline presented on a pocket card) and a plain language summary (summary of the guideline using non-technical language, making it accessible to a wider network of readers, including patients). All documents are publicly available on our website and disseminated through the ERN GENTURIS network and national societies (Table 2).

Three additional clinical guidelines on gastrointestinal stromal tumours, bone sarcomas, soft tissue and visceral sarcomas were developed in collaboration with the European Society for Medical Oncology (ESMO), ERN EURACAN, and ERN PaedCan in accordance with ESMO standard operating procedures for guideline development. Furthermore, ERN GENTURIS endorses existing guidelines if the quality of the guideline is deemed sufficient by at least two independent expert assessors after evaluating the rigor and transparency of the guideline development process. Since the beginning of the network 17 existing guidelines have been endorsed (Table 2).

ERN GENTURIS care pathways describe how to care for patients with a genturis, from identification, diagnosis, and multidisciplinary case discussions to surveillance. Care pathways are guides for healthcare professionals that describe the various tasks involved in the care for patients with a specific condition using evidence-based practice and are intended to help with clinical decision-making. ERN GENTURIS has developed 9 care pathways: *NF2*-related schwannomatosis, non-*NF2* related schwannomatosis, neurofibromatosis type 1, Lynch Syndrome, Hereditary Breast and Ovarian Cancer syndrome, *PTEN* Hamartoma Tumour Syndrome, Li-Fraumeni and heritable -related cancer, Birt-Hogg-Dubé syndrome, Constitutional Mismatch Repair Deficiency. All care pathways developed by ERN GENTURIS are publicly available on the ERN GENTURIS website (Table 2).

## Patient participation and patient journeys

ERN GENTURIS values the patient perspective and patient organisations are encouraged to participate in ERN GENTURIS decision-making processes by becoming a member organisation of the ERN GENTURIS European Patient Advocacy Group (ePAG). Individual genturis patients can also become ePAG representatives (Table 2). ePAG patient representatives liaise with the ePAG member organisation to ensure accurate and equitable representation of the patient voice by participating in the ERN GENTURIS Board, Thematic disease Groups and guideline groups.

**Fig. 2:**
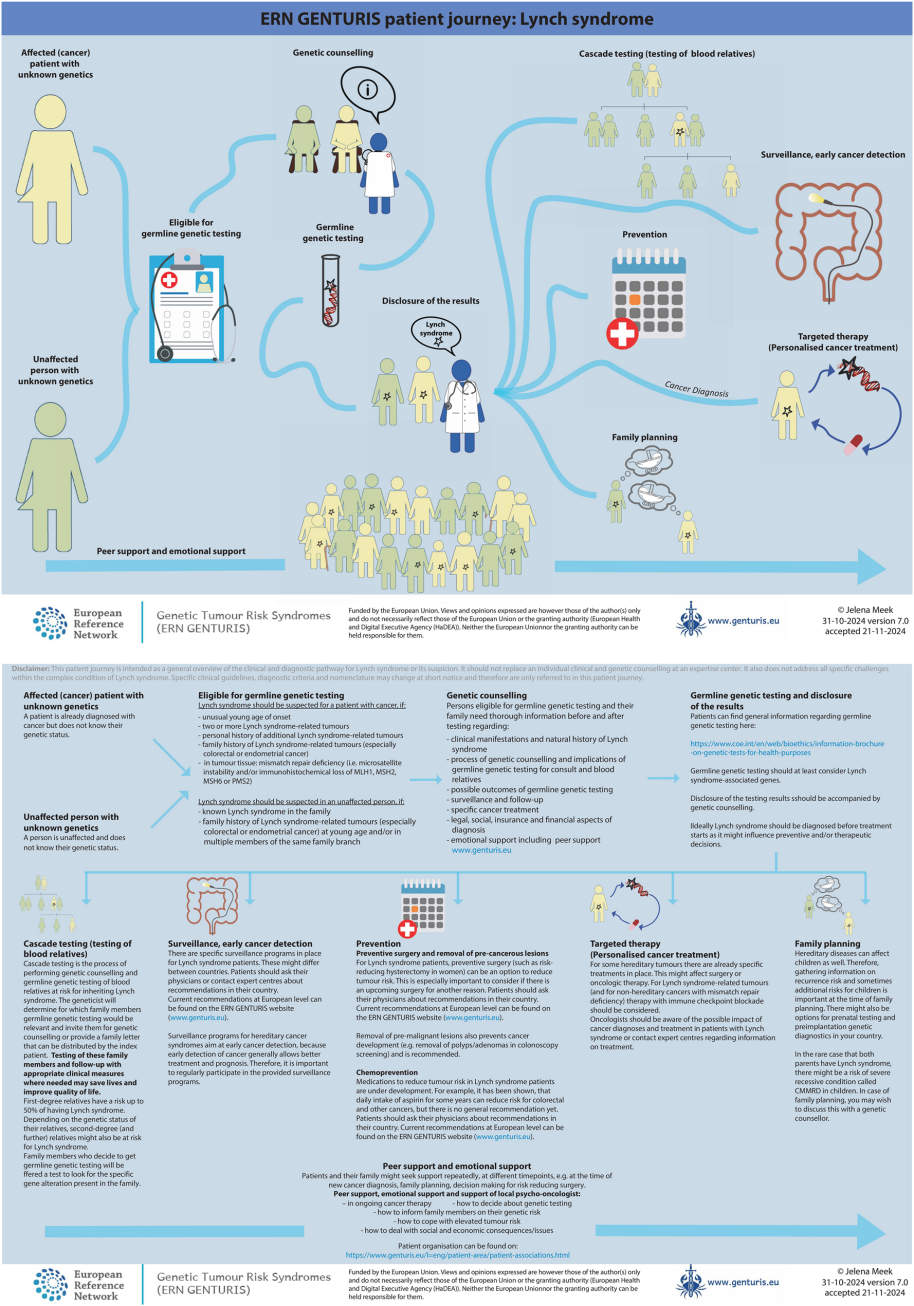
An example of an ERN GENTURIS patient journey: A visual overview (first page) and an information matrix (second page). A full-size image of the patient journey can be found on the ERN GENTURIS website as well: 2024_ERN-GENTURIS_Patient-journey_Lynch-syndrome.pdf (https://www.genturis.eu/l=eng/Assets/2024_ERN-GENTURIS_Patient-journey_Lynch-syndrome.pdf)

In addition to guidelines and care pathways developed for clinicians, ERN GENTURIS also develops patient journeys for patients. These patient journeys provide the patient with a first impression of what to expect. The intended application of the patient journey is for a clinical expert to use the patient journey to explain the route/journey a patient will take. The details of the patient’s personal journey will be filled in by the clinician (personalised care). Each patient journey consists of an accessible visual overview, supported by a detailed information matrix (Fig. 2). ERN GENTURIS has generated 8 patient journeys (neurofibromatosis type 1, *NF2*-related schwannomatosis, non-*NF2*-related schwannomatosis, Lynch syndrome, hereditary breast and ovarian cancer syndrome, *PTEN* hamartoma tumour syndrome, heritable *TP53*-related cancer syndrome/Li Fraumeni syndrome, Birt-Hogg-Dubé syndrome) so far and these are publicly available on our website (Table 2).

## Education

ERN GENTURIS continuously invests in education for both healthcare professionals and the general public by organising regular webinars and courses, and by fostering best practice across Europe. The education programme is meant to increase awareness of genturis and eventually to improve the diagnosis and treatment of genturis thanks to better knowledge of these diseases.

As part of the ERN GENTURIS e-training programme, our webinars provide a general overview of a genturis-related topic or an in-depth look at one of the genturis from a specific healthcare perspective. An important goal of the webinar series is to cover each genturis from several perspectives to create a comprehensive overview of the currently available knowledge for every genturis. The main target audience for the webinars include clinicians, genetic counsellors, clinical scientists, and other health professionals. The webinars last approximately 30–40 min and end with a question & answer session. Over 60 webinar recordings are freely available on the ERN GENTURIS website after registration and can be used as educational material to train students and multidisciplinary team members (Table 2).

ERN GENTURIS also organises several courses to raise awareness about genturis and to specifically train geneticists, oncologists and medical students. The educational course Hereditary Cancer Genetics, organised every two years in collaboration with the European Society of Human Genetics, aims to deliver up-to-date knowledge on hereditary cancers to clinical and molecular geneticists (in training or certified). Another educational course, the Preceptorship on Hereditary Cancer Genetics, is organised every two years in collaboration with the European Society for Medical Oncology. This course provides up-to date knowledge on hereditary cancers to European residents in Medical Oncology. The EJP-RD online course “Diagnosing Rare Diseases: from the Clinic to Research and back”, co-developed by ERN GENTURIS in 2021 is continuously open for enrolment and is intended to help medical students discover the role of research, clinical investigation and data sharing in diagnosing rare diseases. At the national level, ERN GENTURIS members organise courses to share knowledge on genturis.

More information on these courses and registration can be found on the ERN GENTURIS website (Table 2).

## Research and registry

ERN GENTURIS stimulates research on recognition, diagnostics, treatment, and prevention of hereditary cancer by its members to further improve care of patients with a genturis. More information on ongoing and completed ERN GENTURIS research projects, e.g. PREVENTABLE and Solve-RD, as well as an overview of ERN GENTURIS publications can be found on our website (Table 2).

The GENTURIS registry facilitates standardised data registration and sharing of data across Europe to improve diagnosis, treatment and disease prevention in patients with a genturis and contains data of > 1600 patients (as per 13-1-2025).

## Evaluation of the network

The Network and its healthcare providers go through a full evaluation performed by an Independent Evaluation Body (IEB) every five years in line with the Commission Implementing Decision (2014/287/EU) (ERNs Evaluation—European Commission: https://health.ec.europa.eu/rare-diseases-and-european-reference-networks/european-reference-networks/erns-evaluation_en#:~:text=Evaluation%20results%20of%20the%20first%20five%20years%20evaluation,-In%202023%2C%2024&text=100%25%20of%20the%20ERN%20networks,re%2Devaluation%20process%20in%202025.). The IEB checks the operational criteria grouped into seven thematic areas (1. Governance and coordination 2. Clinical care 3. Quality and patient safety 4. Patient centred care 5. Contribution to Research 6. Education and Training 7. Networking and Dissemination), each of which consists of one or more criteria with different measurable elements. According to the Final evaluation report from September 2023, ERN GENTURIS received a 100% score in all measurable elements.

## Conclusion

ERN GENTURIS is a well-established ERN that successfully built a robust infrastructure for healthcare professionals in genetic tumour risk syndromes (genturis, hereditary cancer). ERN GENTURIS facilitates collaboration among healthcare professionals and institutions across Europe, enables exchange of expertise and knowledge via webinars and courses, and develops clinical guidelines and other clinical tools supporting healthcare professionals in their daily work. The network organises regular multidisciplinary patient discussions via the CPMS, has a functioning GENTURIS-registry, numerous research activities, as well as patient empowerment activities. Healthcare providers, patients and families can benefit from these results through improved access to diagnosis, treatment, and the provision of high-quality healthcare to patients with a genturis no matter where they live in Europe. The main achievements and expertise of ERN GENTURIS are publicly available on the ERN GENTURIS website. An overview of specific links on how ERN GENTURIS supports patients, families and healthcare providers is provided in Table 2.

## Data Availability

No datasets were generated or analysed during the current study.

## Footnotes

1 Every year, all European Reference Networks submit the number of new patients referred to their Health Care Providers during the previous year to the European Commission. For ERN GENTURIS centres, this concerns the number of new patients with a (genetically) confirmed diagnosis of a genturis, index and pre-symptomatic.
